# Bending Properties of Carbon Nanotube/Polymer Composites with Various Aspect Ratios and Filler Contents

**DOI:** 10.3390/mi11090857

**Published:** 2020-09-17

**Authors:** Oh-Nyoung Hur, Hyun-Woo Kim, Sung-Hoon Park

**Affiliations:** Department of Mechanical Engineering, Soongsil University, 369 Sangdo-ro, Dongjak-Gu, Seoul 06978, Korea; ohnyung324@soongsil.ac.kr (O.-N.H.); hyunwoi@naver.com (H.-W.K.)

**Keywords:** bending sensor, piezoresistive effect, hysteresis, multi-walled carbon nanotube, aspect ratio

## Abstract

The key characteristics of bending sensors are piezoresistive effect, hysteresis, and durability. In this study, to investigate the influence of the aspect ratio and contents of multi-walled nanotubes (MWNTs) on the properties of bending sensors, MWNT/polydimethylsiloxane (PDMS) composites were fabricated with various aspect ratios and filler contents. The MWNTs were uniformly dispersed in the composites using the three-roll milling method. By increasing the bending angle gradually, the sensitivity of each composite was analyzed. Furthermore, discontinuous cyclic bending tests were conducted to investigate the piezoresistive effect and hysteresis. In addition, stable repeatability of the composites was confirmed through continuous cyclic bending tests. As a result, optimal aspect ratios and filler contents have been presented for application in bending sensors of MWNT composites.

## 1. Introduction

Recently, various studies have been conducted on nanofiller composites for their application in sensors [1,2,3]. Some studies are associated with human motion-detecting sensors, which can detect extremely fine motions such as changes in voice, pulse, and facial expressions as well as large motions such as knee bending and elbow bending [4,5,6,7,8,9]. These sensors are utilized in artificial body parts or flexible robots that can imitate human motion, and in human fitness monitoring systems [10]. Sensors used for these purposes require properties such as long-term stability, fast response, endurance, flexibility, and sensitivity [10,11].

The sensing mechanism of these sensors is the piezoresistive effect, i.e., when compression, tension, or bending is applied to these sensors by human motion, the location and morphology of the fillers in the sensors change, causing a change in electrical resistance. The magnitude of the piezoresistive effect and the electrical properties vary depending on the dimension, content, and morphology of the filler [12,13,14]. In addition, hysteresis may occur due to changes in the position and shape of the fillers in the composite, which are induced by various external deformations [15,16,17]. The piezoresistive effect and the hysteresis phenomenon play an important role in the sensors.

When fabricating nanofiller composites, it is essential to distribute the nanofillers evenly on the polymer composite. If the nanofillers are not uniformly distributed on the composite, the sensing and mechanical properties are degraded with large deviation [18,19,20]. Ultra-sonication method is generally used for nanofiller dispersion [21,22,23] and is appropriate for fabricating low-viscosity composites. However, it is not suitable for producing high-viscosity composites and mass products; therefore, when manufacturing high-viscosity composites, another dispersion method called the three-roll milling method is suggested. The mechanism of three-roll milling involves the dispersion of nanofillers into the composite by the sheer force created between the rolls [24,25]. 

**Figure 1:**
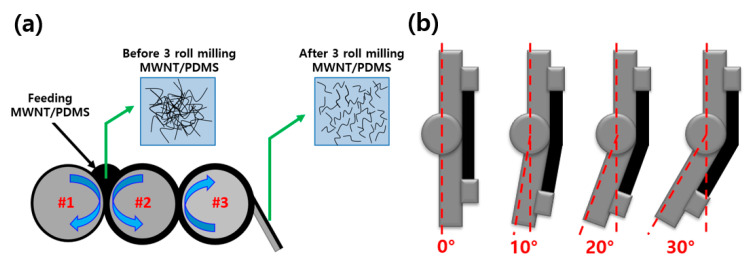
Schematic of (**a**) mechanism for the dispersion of multi-walled nanotubes (MWNTs) in composites by three-roll milling and (**b**) bending process of MWNTs/polydimethylsiloxane (PDMS) composites.

In this study, composites with a similar range of electrical conductivities are fabricated using two types of multi-walled nanotubes (MWNTs) to investigate the variation in bending sensing properties with the change in aspect ratio and the content of MWNTs. Figure 1 shows the schematic of the three-roll milling method and the bending process of MWNT/polydimethylsiloxane (PDMS) composites. Herein, 1 wt.% and 3 wt.% MWNT/PDMS composites were fabricated with low-aspect-ratio MWNTs, and 0.1 wt.% and 1 wt.% MWNT/PDMS composites were fabricated with high-aspect-ratio MWNTs. In the fabrication process, MWNTs were dispersed into the composites using the three-roll milling method, as shown in Figure 1a. Scanning electron microscopy (SEM) was utilized to verify the length of the MWNTs and the degree of dispersion of MWNTs in the composites. In addition, the electrical conductivity and electrical percolation threshold of the composites were measured. Furthermore, the change in electrical resistance was examined while the MWNT/PDMS composite experienced bending as shown in Figure 1b. 

## 2. Materials and Methods

### 2.1. Materials

Two types of MWNTs were used as conducting fillers: 1. The longer one was purchased from JEIO (Incheon, South Korea); it had a mean bundle length 50–150 μm, diameter of 5 nm, and a purity of > 97.5 wt.%; 2. The shorter one was bought from KB-Element (Gyeonggi-do, South Korea), which had an average diameter of 5 nm, length of 10–20 μm, and a purity of > 98 wt.%. Further, PDMS (Dow Corning, Midland, MI, USA) and silver paste (Protavic, Levallois-Perret, France) were used as the base matrix and electrode, respectively. 

### 2.2. Preparation of MWNT/PDMS Composites

For a uniform dispersion of the filler into the matrix, a paste that preferentially comprises PDMS and MWNT was prepared using a paste mixer (Daehwa, Seoul, South Korea) and a three-roll miller (Intech, Gyeonggi-do, South Korea), before hot-press heating stage. Initially, the PDMS base and PDMS curing agent were prepared with a mass ratio of 10:1; then, MWNT was added to it. Next, it was mixed for 30 s at 500 rpm and for 60 s at 1500 rpm using the paste mixer. Finally, to evenly disperse the MWNTs into the PDMS, three-roll milling was performed for 5 min. To estimate the electrical characteristics, composite films of thickness 1 mm were fabricated by curing the paste using a hot-press heating plate (Qmesys Inc., Gyeonggi-do, South Korea) at 150 °C and 15 MPa for 50 min. 

### 2.3. Characterization

To estimate the length of MWNTs, a small amount of MWNTs was dispersed in chloroform (Daejung, Seoul, South Korea) by ultra-sonication. The suspension was then dropped onto a Si wafer and was immediately spin-coated in steps. First, it was spin-coated at 500 rpm for 5 s, followed by 2000 rpm for 30 s, and 500 rpm for 5 s. Finally, it was dried at room temperature for one day. These samples were observed by SEM (Gemini-SEM 300, ZEISS, Land Baden-Württemberg, Germany) to analyze the dispersion degree of the MWNTs in the composites. To observe the cross-section of the composites, they were broken in liquid nitrogen. 

To study the electrical characteristics and the electrical percolation thresholds, MWNT/PDMS composites of different contents were fabricated. To calculate the average value, five specimens were fabricated and tested. The specimen volume was 50 mm × 5 mm × 1 mm. To improve the electrical contact between the electrodes and the specimens, the surface of the specimens was treated by ultraviolet (UV) light irradiation. The samples were irradiated with UV for 300 s with a UV O3 machine (JSE, Seoul, South Korea). Then, to prepare the electrodes, Ag paste was applied to both edges of the specimen surface. The electrodes were cured in an oven at 120 °C for 1 h. The specimens were then cooled at room temperature for one day. Their electrical resistance was measured by a DMM 7510 multimeter (Keithley, Cleveland, OH, USA) using a 2-way wire. 

To investigate the sensitivity of the MWNT/PDMS composites, step bending was performed with a custom-made bending machine (NAMIL, Incheon, South Korea), and the resistance was simultaneously recorded using the DMM 7510 multimeter. The change in electrical resistance was measured by increasing the bending angle up to 30° in steps of 3°. After each step, a stabilization time of 20 s was allowed. The electrical resistance at each angle was obtained by averaging 20 values during the stabilization time. 

To understand the hysteresis, specimens were bent up to 30° from the steady state with an increase of 10° three times; a stabilization time of 30 s was used in each step. After bending up to 30°, the specimens were released to the steady state with a decrease of 10° three times, and each step had a stabilization time of 30 s. When they were bending or decreasing, the bending rate was 1°/sec. As the bending angle of the MWNTs/PDMS composite was changed, the electrical resistance was simultaneously measured using the DMM 7510 multimeter. Furthermore, to comprehend stable repeatability, the MWNTs/PDMS composite, which was already pre-bent 15 times up to 30°, was subjected to continuous bending and releasing for 100 cycles up to 30°.

## 3. Results and Discussion

### 3.1. Morphology Analysis

**Figure 2:**
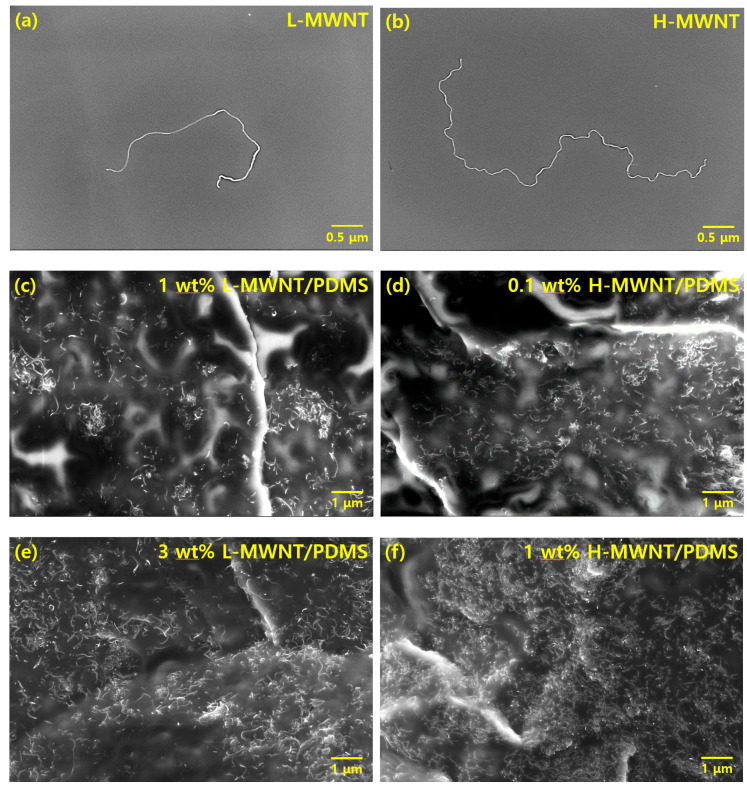
SEM images of MWNTs: (**a**) low-aspect-ratio MWNT (L-MWNT); (**b**) high-aspect-ratio MWNT (H-MWNT); and cross-section of MWNTs/PDMS composites: (**c**) 1 wt.% L-MWNT/PDMS; (**d**) 0.1 wt.% H-MWNT/PDMS; (**e**) 3 wt.% L-MWNT/PDMS; and (**f**) 1 wt.% H-MWNT/PDMS.fref.

The SEM images (Figure 2) were obtained to determine the aspect ratio of MWNTs and confirm whether the MWNTs were well-dispersed in the composite. Several SEM images were obtained to determine the mean length of each MWNT and confirm the degree of dispersion. The MWNT shown in Figure 2a has an average length of 4.5 μm and a diameter of 5 nm; therefore, its aspect ratio is 900. We refer to this MWNT as the low-aspect-ratio MWNT (hereinafter referred to as L-MWNT). The MWNT shown in Figure 2b has a mean length of 12 μm and a diameter of 5 nm; therefore, its aspect ratio is 2400. This MWNT was named as the high-aspect-ratio MWNT (hereinafter referred to as H-MWNT). By comparing Figure 2a with Figure 2b, it can be observed that the H-MWNT has a more prominent winding shape than L-MWNT. 

Figure 2c–f show the SEM images of the cross-section of the MWNTs/PDMS composite. From Figure 2c–f, it can be confirmed that both L-MWNT and H-MWNT are individually well-dispersed in the composite without agglomerates regardless of the filler content. Comparing Figure 2c with Figure 2f, more H-MWNTs were observed than L-MWNTs at the same content. This is because MWNTs with larger aspect ratio have a higher probability of being observed in the polymer matrix. 

### 3.2. Electrical Conductivity and Percolation Threshold

**Figure 3:**
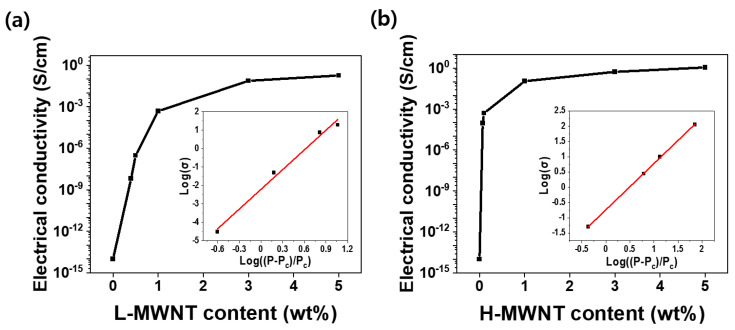
Electrical conductivity versus MWNT content: (**a**) L-MWNT/PDMS composite and (**b**) H-MWNT/PDMS composite. The insets in the figures refer to the log(σ) value versus log((P − P_c_)/P_c_).

The electrical characteristics of MWNTs composites are principally related to the morphology and content of the MWNTs. Figure 3 depicts the variation of the electrical conductivity and electrical percolation threshold values with different MWNTs contents and aspect ratios. The electrical conductivity of the MWNTs/PDMS composites was determined by the following equation:

σ_ec_ = L/(A_c_R_e_) (1)

where σ_ec_ represents electrical conductivity, L denotes the distance between the two electrodes, A_c_ denotes the cross-sectional area of the sample, and R_e_ is the electrical resistance of the specimen.

As shown in Figure 3a,b, the electrical conductivity increases as the content of MWNTs increases and saturates above a certain content. As the amount of filler increases, the number of electrical networks in the composite increases, thereby increasing the electrical conductivity of the composite. However, if the amount of filler exceeds a certain value, the electrical networks saturate inside the composite, and the electrical conductivity does not increase drastically with the increasing filler content. Furthermore, the electrical conductivity of H-MWNT/PDMS is higher than that of L-MWNT/PDMS for the same wt.% of filler. This is because H-MWNT has a larger aspect ratio than L-MWNT; therefore, it is more advantageous for forming electrical networks inside composites [26,27].

The following equation was used to determine the electrical percolation threshold of each MWNTs/PDMS composite [28,29].

σ ∝ (P − P_c_)^k^ (2)

where σ is the electrical conductivity at the filler volume P, P_c_ denotes the filler volume at the percolation threshold, and k is the critical exponent. In the inset images of Figure 3a,b, the slope of the red line means k factor. As calculated using Equation (2), P_c_ and k for the L-MWNT/PDMS composite are 0.4 wt.% and 3.56, respectively, and those for the H-MWNT/PDMS composite are 0.07 wt.% and 1.52, respectively. The P_c_ value of the L-MWNT/PDMS composite is larger than that of the H-MWNT/PDMS composite, because of the difference in the aspect ratios of MWNTs in each composite [27,30,31]. Because the H-MWNT has a larger aspect ratio than L-MWNT, a sufficient number of electrical networks are formed, thus allowing electricity to flow inside the composite even if only smaller amounts of filler are used.

### 3.3. Bending Sensing Properties

#### 3.3.1. Sensitivity

**Figure 4:**
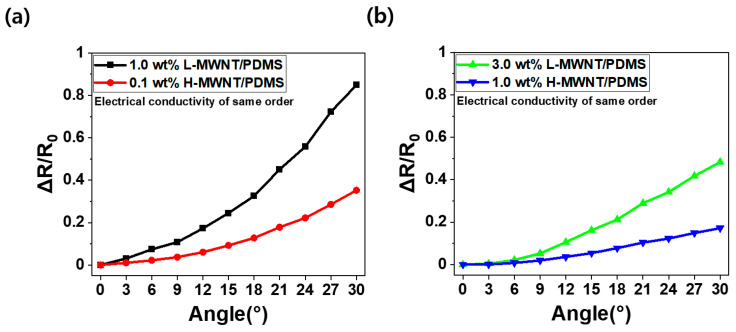
ΔR/R_0_ according to bending angle: (**a**) 1.0 wt.% L-MWNT/PDMS (black) and 0.1 wt.% H-MWNT/PDMS (red). (**b**) 3.0 wt.% L-MWNT/PDMS (green) and 1.0 wt.% H-MWNT/PDMS (blue).

The electrical conductivities of 1 wt.%, 3 wt.% L-MWNT/PDMS, and 0.1 wt.%, 1 wt.% H-MWNT/PDMS obtained using Equation (1) are 0.05 S/m, 7.20 S/m, and 0.05 S/m, 9.76 S/m, respectively. To compare the bending sensing properties of the MWNTs/PDMS composites, considering the aspect ratios of MWNTs in a similar range of electrical conductivity, 1 wt.% L-MWNT/PDMS and 0.1 wt.% H-MWNT/PDMS are chosen as a comparative group. Similarly, 3 wt.% L-MWNT/PDMS and 1 wt.% H-MWNT/PDMS are selected as a comparison group. Figure 4 shows the ΔR/R_0_ value of each composite according to the change in the bending angle; here, R denotes real-time electrical resistance and R_0_ represents initial state electrical resistance. 

Figure 4a shows the rate of change of R with respect to R_0_ (ΔR/R_0_) over a range of bending angle and aspect ratio of the MWNTs. In Figure 4a,b, ΔR/R_0_ value increased non-linearly with the increase of the bending angle. It is because the MWNT electrical networks inside the composite experience more disconnected networks as the bending deformation increases. In addition, L-MWNT was more sensitive than H-MWNT. The electrical networks in the L-MWNT composite were easily broken even if the same bending deformation was applied, owing to the smaller aspect ratio of the L-MWNT composite; this made them more sensitive to bending.

The rate change of the ΔR/R_0_ value with respect to the strain is called the gauge factor or sensitivity [32,33]. For the bending sensor considering the bending angle instead of the strain, i.e., the rate change of ΔR/R0 value with respect to the bending angle is a similar concept to the gauge factor. It is 3%/1° for 1.0 wt.% L-MWNT/PMDS, 1%/1° for 0.1 H-MWNT/PDMS, 2%/1° for 3.0 wt.% L-MWNT, and 0.6%/1° for 1.0 wt.% H-MWNT/PDMS, respectively. These results indicate that L-MWNT/PDMS composite was more sensitive to bending deformation than the H-MWNT/PDMS composite. In addition, the sensitivity decreased as the MWNT content increased. This is because as the MWNT content increased, the MWNT electrical networks inside the composite were disconnected only by a small portion due to excessive number of MWNTs [34,35]. Furthermore, the sensitivity of 1.0 wt.% H-MWNT/PDMS was greater than that of 1.0 wt.% L-MWNT/PDMS at the same content. This is because the aspect ratio of L-MWNT is smaller than that of H-MWNT, resulting in increased disconnections of the electrical networks in the MWNTs according to the change [15,35]. Therefore, a smaller aspect ratio of MWNT and decreased content (over P_c_) are appropriate conditions for an MWNTs/PDMS composite bending sensor for improving the sensitivity.

#### 3.3.2. Piezoresistive Effects in Cyclic Bending

**Figure 5:**
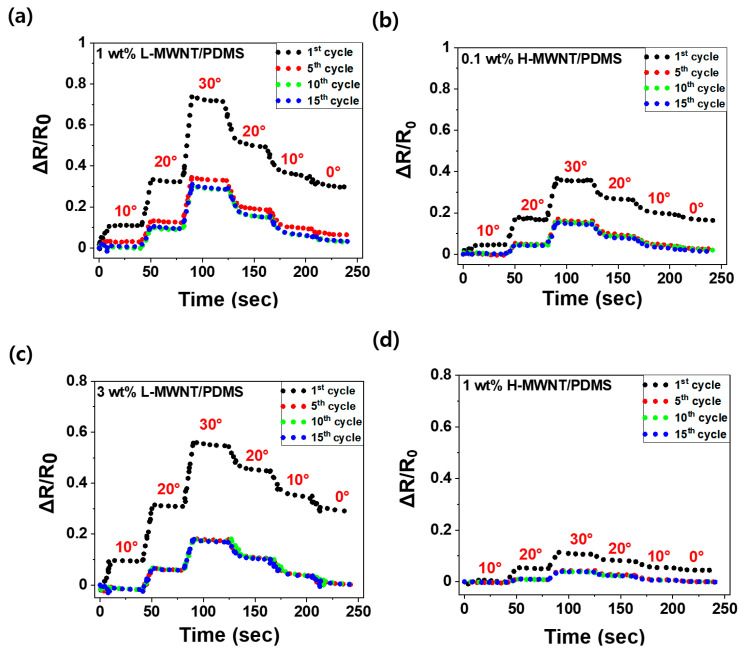
ΔR/R_0_ value according to bending angle for MWNT/PDMS composites under discontinuous 15 cyclic bending and releasing deformations: (**a**) 1 wt.% L-MWNT/PDMS, (**b**) 0.1 wt.% H-MWNT/PDMS, (**c**) 3 wt.% L-MWNT/PDMS, (**d**) 1 wt.% H-MWNT/PDMS. (red text: bending state of each angle).

Figure 5 shows the change in ΔR/R_0_ value under 15 discontinuous bending and releasing deformations. In all samples, the ΔR/R_0_ value increased when the samples were bent and decreased when the samples were released; this phenomenon is called the piezoresistive effect. Moreover, ΔR/R_0_ did not return to its original value when the samples were bent up to 30° and then returned to 0°; this phenomenon is called hysteresis on piezoresistance. Hysteresis is a condition in which a physical state depends on the process it underwent. For example, as the MWNTs/PDMS composite underwent bending and releasing processes, permanent deformation of the MWNT networks inside the composite occurred; therefore, ΔR/R_0_ could not return to its original value. In other words, if the bending and releasing actions are repeated, the position of the MWNTs in the composite varies and the MWNTs buckle because they undergo repeated bending and releasing deformation. Therefore, the electrical networks inside the composites are reshuffled, causing hysteresis [15].

The hysteresis phenomenon can be mitigated by repeating the sensor bending and releasing motions several times. Figure 5a,b show that the hysteresis value of 1 wt.% L-MWNT/PDMS decreased from 29% in the 1st cycle to 3.2% in the 15th cycle, and that of 0.1 wt.% H-MWNT/PDMS decreased from 16% to 1.3%. Figure 5c,d show that the hysteresis value of 3 wt.% L-MWNT/PDMS decreased from 28% in the 1st cycle to 0.2% in the 15th cycle, and that of 1 wt.% H-MWNT/PDMS decreased from 4.3% to 0%. The hysteresis values of all samples saturated to a sufficiently small value of 0–3% when the MWNT/PDMS composites underwent bending and releasing deformations after 10–15 cycles.

Apart from a decrease in the hysteresis value, the ΔR/R_0_ value also decreased following repeated bending and releasing deformations. Figure 5a,b show that, at a bending angle of 30°, the ΔR/R_0_ value of 1 wt.% L-MWNT/PDMS decreased from 74% in the 1st cycle to 31% in the 15th cycle, and that of 0.1 wt.% H-MWNT/PDMS decreased from 36% to 15%. Figure 5c,d show that, at a bending angle of 30°, the ΔR/R_0_ value of 3 wt.% L-MWNT/PDMS decreased from 55% in the 1st cycle to 17% in the 15th cycle, and that of 1 wt.% H-MWNT/PDMS decreased from 11% to 5.0%.

Here, both ΔR/R_0_ and the hysteresis values decreased and ultimately saturated on repeating the bending and releasing deformations after approximately 15 cycles. This can be explained by the rearrangement and buckling of MWNTs in the composite induced by repeated bending and releasing deformations. Therefore, when MWNTs/PDMS composites are used as bending sensors, a pre-bending for 10–15 times is necessary to obtain constant data even with repeated bending. Further, because the ΔR/R_0_ value decreases due to repeated bending and releasing deformations, L-MWNT/PDMS composites with relatively large ΔR/R_0_ values even after pre-bending are more appropriate as bending sensors than H-MWNT/PDMS composites, in terms of hysteresis.

**Figure 6:**
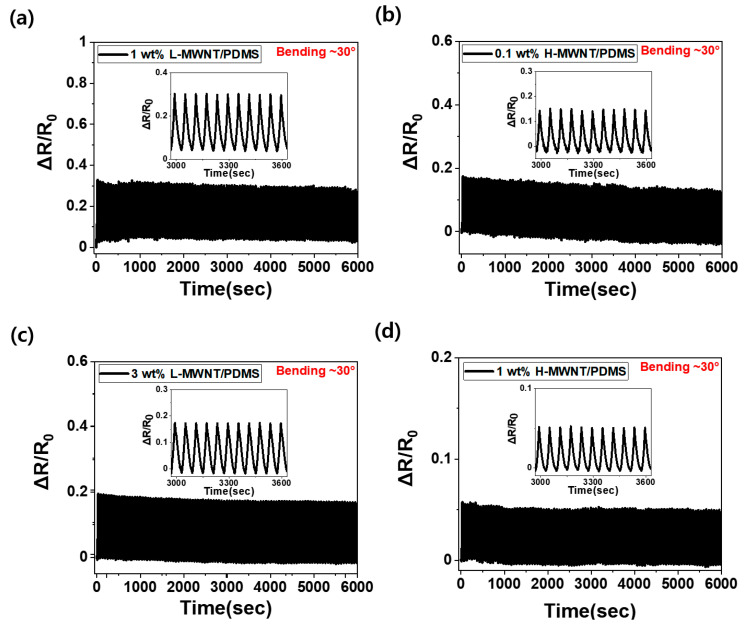
ΔR/R_0_ following 100 cycles of continuous bending and releasing deformations up to a bending angle of 30°: (**a**) 1 wt.% L-MWNT/PDMS; (**b**) 0.1 wt.% H-MWNT/PDMS; (**c**) 3 wt.% L-MWNT/PDMS; (**d**) 1 wt.% H-MWNT/PDMS. The inset image is an enlarged image of the 50–60 cycles.

In bending sensors, it is important to measure the signals stably even with repeated bending. Figure 6 depicts the measurement of stable ΔR/R_0_ values even with 100 continuous bending (up to 30°) and releasing cycles. As observed from each figure, the section where the ΔR/R_0_ value increases is in the bending state, and the section where the ΔR/R_0_ value decreases is in the releasing state. As shown in Figure 6a–d, stable ΔR/R_0_ values are obtained even with 100 continuous bending and releasing cycles, for both 1 wt.%, 3 wt.% L-MWNT/PDMS, and 0.1 wt.%, 1 wt.% H-MWNT/PDMS. The ΔR/R_0_ values appeared to be stable even when the bending sensors were subjected to repeated bending and releasing because the sensors were pre-bent 15 times (up to 30°) before being used for bending sensing. The rearrangement and buckling phenomena of the MWNTs inside the composite were already saturated when they were pre-bent 15 times before subjecting to 100 continuous bending and releasing deformation cycles. Therefore, even in repeated bending and releasing cycles, constant ΔR/R_0_ values can be obtained.

A comparison of Figure 6a with Figure 6c, and Figure 6b with Figure 6d, indicates that the ΔR/R_0_ value becomes smaller as the MWNT content increases during continuous cyclic bending and releasing deformations. Furthermore, a comparison of Figure 6a and Figure 6d indicates that the 1 wt.% L-MWNT/PDMS composite is more sensitive to bending than the 1 wt.% H-MWNT/PDMS composite under continuous cyclic bending and releasing deformations. The L-MWNT/PDMS composite is more sensitive than the H-MWNT/PDMS composite even at the same content because of the larger aspect ratio and wavy shape of the H-MWNT. The large aspect ratio and the wavy shape played a key role in preventing disconnections of the MWNT networks inside the composite when subjected to bending deformation, thereby mitigating the piezoresistive effect [15,35].

## 4. Conclusions

The bending sensing properties of MWNT/PDMS composites, which vary depending on the aspect ratio and content of the filler, were investigated. To determine the influence of aspect ratio and the content of the MWNTs on the sensitivity of the bending sensor, each composite was bent up to 30° in steps of 3°, and the rate of change of ΔR/R_0_ value was investigated. It was confirmed that the sensitivity was better as the aspect ratio was smaller and the content of MWNT decreased. Furthermore, the results of discontinuous cyclic indicated that pre-bending for more than 15 times is necessary for ensuring stable bending sensing. Finally, continuous cyclic bending and releasing tests were performed to verify that the bending sensor’s durability is reliable with maximum sensitivity. It was demonstrated that all the pre-bent composites obtained a stable ΔR/R_0_ values. Therefore, the composite having a smaller aspect ratio and lesser content of MWNTs is optimal for the properties of bending sensors. The results of this study provide insights for designing an MWNT/PDMS composite that can be utilized in motion bending sensors.
